# Alfredson versus Silbernagel exercise therapy in chronic midportion Achilles tendinopathy: study protocol for a randomized controlled trial

**DOI:** 10.1186/s12891-017-1656-4

**Published:** 2017-07-11

**Authors:** Bas Habets, Robert E. H. van Cingel, Frank J. G. Backx, Bionka M. A. Huisstede

**Affiliations:** 1 Papendal Sports Medical Center, Papendallaan 7, 6816 VD Arnhem, The Netherlands; 2 Research Institute for Health Sciences, IQ Healthcare, Radboud University Medical Center, P.O. Box 9101, 6500 HB Nijmegen, The Netherlands; 3 Department of Rehabilitation, Physical Therapy Science and Sports, Rudolf Magnus Institute of Neurosciences, University Medical Center Utrecht, P.O. Box 85500, Utrecht, 3508 GA The Netherlands

**Keywords:** Achilles tendinopathy, Achilles tendon, Exercise therapy, Eccentric, Concentric, Physical therapy, Physiotherapy, Rehabilitation

## Abstract

**Background:**

Midportion Achilles tendinopathy (AT) is a common overuse injury, usually requiring several months of rehabilitation. Exercise therapy of the ankle plantar flexors (i.e. tendon loading) is considered crucial during conservative rehabilitation. Alfredson’s isolated eccentric and Silbernagel’s combined concentric-eccentric exercise programs have both shown beneficial results, but it is unknown whether any of these programs is superior for use in clinical practice. Therefore, the primary objective of this study is to compare the effectiveness of both programs on clinical symptoms. Secondary objectives are to compare the effectiveness of both programs on quality of life and functional outcome measures, to investigate the prognostic value of baseline characteristics, to investigate differences in cost-effectiveness.

**Methods/Design:**

Eighty-six recreational athletes (21–60 years of age) with unilateral chronic midportion AT (i.e. ≥ 3 months) will be included in this multicenter assessor blinded randomized controlled trial. They will be randomly allocated to either a group performing the Alfredson isolated eccentric training program (*n* = 43), or a group performing the Silbernagel combined concentric-eccentric program (*n* = 43). In the Alfredson group, participants will perform eccentric heel-drops on their injured side, twice daily for 12 weeks, whereas in the Silbernagel group, participants perform various concentric-eccentric heel-raise exercises, once daily for 12 weeks. Primary outcome measure will be the Victorian Institute of Sport Assessment – Achilles (VISA-A) questionnaire. Secondary outcomes will be a visual analogue scale (VAS) for pain during daily activities and sports, duration of morning stiffness, global perceived effect, the 12-item Short Form Health Survey and the Euroqol instrument, and functional performance measured with the heel-raise test and the countermovement jump. Additionally, alongside the RCT, a cost-effectiveness analysis will be performed. Assessments will be performed at baseline and after 12, 26, and 52 weeks.

**Discussion:**

This study is the first to directly compare the Alfredson and the Silbernagel exercise program in a randomized trial. The results can further enlarge the evidence base for choosing the most appropriate exercise program for patients with midportion AT.

**Trial registration:**

Dutch Trial register: NTR5638. Date of registration: 7 January 2016.

## Background

Midportion Achilles tendinopathy (AT) is a common overuse injury of the lower extremity, [1, 2] most prevalent in male athletes who participate in sports that involve running and/or jumping. [2–4] When not adequately managed, the injury may cause long term absenteeism of sports and daily activities. [5] Treatment of midportion AT is initially conservative, usually requiring several months, with a plethora of possible treatment options. [6, 7].

Historically, AT is considered as an inflammatory condition, but more recently it has been regarded as a failed healing response of the tendon, with minimal inflammatory influence. [8, 9] In 2009, Cook and Purdam proposed a model that considers tendinopathy as a continuum, in which three somewhat interchangeable stages can be distinguished: 1) reactive tendinopathy, 2) tendon dysrepair, and 3) degenerative tendinopathy. [10] According to the authors, these stages all require tailored load management and exercise intervention strategies. The model was recently revisited, [11] but it is still generally agreed that exercise therapy (i.e. tendon loading) is crucial to promote improvement of symptoms and function. [3, 9, 12].

Several exercise programs have shown favourable results in mid-portion AT, with both beneficial effects on pain and function. Recent studies concluded that there is strong evidence for eccentric exercise therapy, [6, 7] particularly according to the Alfredson eccentric exercise program. [13] In the Alfredson program, the plantar flexor muscle-tendon unit is loaded eccentrically by performing heel drops on the injured side, while using the non-injured limb to (concentrically) return to the start position. [14] A total of 180 repetitions is performed daily, and this may be a great time-consuming burden for the patient, potentially compromising compliance and consequently the effectiveness of the program. Although the majority of studies using the Alfredson program reported significant improvements post-intervention, [14–17] it should be noted that other studies reported less positive effects. [18, 19] Moreover, a recent study of Stevens & Tan (2014) showed that a less stringent “do-as-tolerated” eccentric protocol can lead to equal improvements in pain and function compared to the Alfredson protocol, [20] which may be advantageous from a patient perspective. However, as exercises were performed only for a period of 6 weeks, and mid- and long-term follow-up measurements (i.e. > 6 weeks) were lacking, conclusions should be interpreted with caution.

Also, exercise programs other than isolated eccentric loading showed to be effective in AT. [21, 22] In a recent randomized controlled trial (RCT), Beyer et al. [23] found that heavy slow resistance training (HSRT) using gym equipment leads to equally good clinical improvement compared with the Alfredson program. Furthermore, in an earlier systematic review, Malliaras et al. [24] already concluded that there is equivalent evidence for the Silbernagel concentric-eccentric exercise program, although this conclusion was based on limited evidence. Unlike the Alfredson protocol, the Silbernagel protocol also comprises concentric and even plyometric loading of the Achilles tendon. [25, 26] From a patient perspective, a potential benefit of the Silbernagel program over the Alfredson program, may be the frequency of the exercises (i.e. only once a day). This may encourage training compliance and consequently can result in better outcomes. Furthermore, a combination of concentric and eccentric loading may better restore concentric muscular deficits, as training gains are known to be specific to the contraction mode. [27].

Although both the Alfredson and Silbernagel program have shown favorable results in midportion AT, [14, 16, 17, 25, 26, 28, 29] comparison of the results is hampered by heterogeneity of study populations. [13, 24] Insight into whether one of these programs is superior may lead to better results in the management of patients with AT.

This article provides a detailed description of the study design, target population, and methods/procedures of a pragmatic multicenter RCT that will investigate differences in effectiveness between the Alfredson and Silbernagel exercise program for patients with midportion AT.

### Study objectives

The primary objective of this study is to compare the effectiveness in terms of symptom reduction and function of the Alfredson isolated eccentric exercise program to the Silbernagel concentric-eccentric exercise program after 12 months in patients with chronic midportion AT.

Secondary objectives are 1) to investigate differences in effectiveness on global perceived effect and quality of life (QOL), 2) to investigate differences in effectiveness on functional outcome measures, and 3) to investigate the prognostic value of baseline characteristics. Furthermore, alongside this RCT, a cost-effectiveness evaluation between both programs will be performed.

## Methods/Design

### Study design and setting

This protocol was developed in accordance with the SPIRIT guidelines, [30] and describes an assessor blinded multicenter parallel-group RCT, with a one-year follow-up. The study will be conducted in two different centers, i.e. the University Medical Center Utrecht (UMCU, department of Rehabilitation, Physical Therapy Science & Sports), Utrecht, The Netherlands, and Papendal Sports Medical Center, Arnhem, The Netherlands. Participants will be randomized to a group performing either the Alfredson isolated eccentric or the Silbernagel combined concentric-eccentric exercise program. Randomization will be performed using a web-based randomization system, and allocation will be concealed. The investigators who are involved in baseline and follow-up measurements, and data analysis will be blinded to group allocation.

**Fig. 1:**
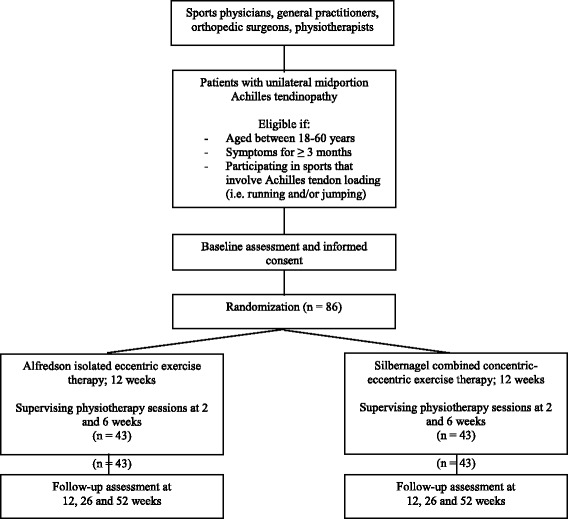
Flow chart of the study design

Measurements will be performed at baseline, and after 12, 26, and 52 weeks follow-up (see Fig. 1). The study protocol is in accordance with Declaration of Helsinki, and has been approved by the ethics committee of the UMCU (registration number 16–158). The protocol was registered with the Dutch Trial Register on 7 January 2016 (NTR5638). Written informed consent will be obtained from all participants prior to their participation.

### Participant selection

Recreational athletic patients (both male and female) with a clinical diagnosis of unilateral midportion AT, characterized by activity-related Achilles tendon pain and swelling at 2 to 7 cm from the calcaneal insertion, [31] are eligible for inclusion if they meet the following inclusion criteria: 1) 18–60 years of age, 2) duration of symptoms of at least 3 months, 3) participating in sports involving Achilles tendon loading (i.e. sports characterized by walking, running and/or jumping), and 4) able to comply with both exercise programs.

Participants are excluded in case of: 1) bilateral symptoms, 2) diagnosis of insertional AT, 3) washout period of <4 weeks from other treatments for their AT, 4) corticosteroid injections in the region of the Achilles tendon in the previous 12 months, 5) other lower limb injuries of the affected limb in the previous 12 months, 6) musculoskeletal surgery of the affected limb in the previous 12 months, 7) history of Achilles tendon rupture in the affected limb, or 8) systemic diseases, such as rheumatoid arthritis or diabetes mellitus.

### Sample size calculation

Sample size was calculated based on two high quality RCTs having investigated the Alfredson program and the Silbernagel program respectively, and using the VISA-A questionnaire as their primary outcome measure. [16, 26] We used the respective change (mean ± standard deviation [SD]) in VISA-A scores for the groups that followed the above-mentioned exercise programs:

- Rompe et al. [16] (*n* = 25): VISA-A Δ 22.4 ± 19 for the Alfredson program
- Silbernagel et al. [26] (*n* = 19): VISA-A Δ 34 ± 17 for the Silbernagel program

This resulted in an expected effect size of 0.64 between both exercise programs, with an expected VISA-A change score that exceeds the minimal clinically important difference of 10 points. [23] Using G*Power 3.1, and assuming α two-sided = 0.05 and a power of 0.80, a total of 39 participants in each study arm was required. The dropout rate in the above-mentioned studies was 4% [26] and 9% [16] respectively. We chose to take the most conservative dropout rate of 9% into account, resulting in a required amount of 86 in total, i.e. 43 participants in each arm.

### Recruitment and informed consent

Primarily, participants are recruited from the patient population of the two afore mentioned centers. Secondary, general practitioners and orthopedic surgeons in the surroundings of Papendal Sports Medical Center (Arnhem, The Netherlands), will be asked to identify participants.

Eligible participants will be informed about the study by their treating (sports) physician or physiotherapist through an information letter, and they will initiate contact with the coordinating investigator (BH). Eligibility criteria of these participants will initially be checked by telephone, and subsequently, if they meet the criteria, an appointment is made for baseline assessment. Prior to baseline assessment, eligibility criteria will be confirmed and participants will sign informed consent.

### Randomization procedure

Randomization will be performed directly after baseline assessment, by an independent secretary using a computer-generated random sequence table. Eighty-six envelopes will be prepared with a description of the allocated intervention (i.e., Alfredson or Silbernagel program). These envelopes will be sealed, and then shuffled and sequentially numbered. After baseline assessment, the secretary will pick an opaque sealed envelope according to the randomization table. Subsequently, within 1 week participants will be referred to one of the supervising physiotherapists, who is informed about the allocated program by the independent secretary. During the first session, participants will receive detailed instructions on the allocated exercise program.

The randomization code will not be broken until the final follow-up measurement has been performed (i.e. participant’s last visit), and data analysis has been completed. Participants are instructed not to reveal their group allocation to the investigator during all measurement procedures.

### Intervention

One study arm performs the Alfredson isolated eccentric exercise program, [14] which comprises 12 weeks of eccentric heel-drops on the injured limb, with the use of the uninjured limb to concentrically return to the start position. Exercises are performed twice daily, for three sets of 15 repetitions, both with a straight and bent knee (i.e. 180 repetitions each day). Non-disabling pain during the exercises is permitted, and load is added gradually in a backpack (in steps of 5 kg) when exercises can be performed without pain.

The second study arm performs an exercise program according to the Silbernagel protocol. [25, 26] This program comprises various concentric and eccentric heel raise exercises, which are performed both on two legs and one leg, with three sets of 15 repetitions. The duration of the program is also 12 weeks, and non-disabling pain during the exercises is also permitted, but contrary to the Alfredson program, exercises are performed only once daily. Progression is made by changing from bipedal to unipedal exercises, by progressing from concentric-eccentric to purely eccentric loading, by adding weight in a backpack (in steps of 5 kg when pain did not exceed 5 on a 0–10 numerical rating scale), and finally by using fast-rebounding and plyometric exercises.

Table 1 depicts the key features of both exercise programs. The timing of the exercise as well as the time under tension are not described, as we wanted to replicate the clinical prescription of the exercise programs.

**Table 1:** Key features of the exercise programs

	Alfredson	Silbernagel
Duration of exercise program	12 weeks	12 weeks
Frequency of exercises	Twice daily	Once daily
Amount of exercises	2	4–5
Sets and repetitions	3 × 15	3 × 15
Exercise mode	Slow isolated eccentric	Concentric, eccentric, plyometric
Pain tolerated	Non-disabling pain	Not more than 5 on a 0–10 NRS
Progress	No pain	Phase 1–2 – 3
Progression	Add load (5 kg)	Add load (5 kg)

*NRS* numerical rating scale

The content of both programs will be instructed in detail by the supervising physiotherapists during the first appointment. Participants will perform all exercises from both programs at home. After two and 6 weeks of training, an appointment with the supervising physiotherapist is made to discuss potential difficulties with the exercises and adjust load when possible.

During the intervention period, participants are asked to refrain from other treatments and from anti-inflammatory medication related to their injury. If they receive other (medical) treatments after the intervention period, they are asked to register this in a logbook.

Participants in both study arms are advised not to participate in any tendon loading sports activities (i.e. walking, running and jumping) during the first 3 weeks of the intervention period. [23] Subsequently, they are allowed to resume tendon loading sports activities, as long as pain does not exceed 50 mm on a 0–100 mm visual analogue scale (VAS), and pain subsides within 24 h after the activity. [26].

### Education and monitoring

The research team will organize an information meeting in order to inform the involved physiotherapists of the two participating centers about the study procedures, their exact role, and the content of the exercise programs. During this meeting, written information is also provided. For potential referrers, an information letter will be sent by the coordinating investigator. In this letter, the objectives of the study and a short description of the study design are described.

Monitoring of study procedures will be performed by an independent monitor during multiple visitations. These include an inspection of the study file for each center prior to the start of the study, and a check of the procedures and study files after 1 year and at the end of the study.

### Outcome measures

#### Baseline assessment

During baseline assessment – besides the primary, secondary and other outcome measures – demographic and anthropometric characteristics such as age, weight, height, body mass index, job type and activity level, sport type and activity level, and referral type are recorded using a standardized questionnaire. Additionally, waist circumference will be recorded with a flexible tape measure, [32] range of motion for ankle dorsiflexion will be recorded using the weight bearing lunge test, [33] and dorsiflexion range of motion of the first metatarsophalangeal joint will be measured with a standard goniometer. [34] Body weight and sport activity level will also be recorded at T1, T2 and T3, as these variables are thought to vary throughout the study period and thus may potentially influence the study outcome.

#### Primary outcome measure

The primary outcome for this study will be the difference in VISA-A scores between both programs after 12 months. The VISA-A questionnaire has been shown reliable and valid for evaluating clinical severity of symptoms of AT, [35] and was recently translated/validated in Dutch. [36] It consists of eight questions, covering the three domains of pain, and function in daily living and sporting activities. Scores range from 0 to 100, where 100 represents a perfect function.

#### Secondary outcome measures

A VAS will be used to evaluate severity of pain during sports and daily activities for the past 7 days. The VAS is a 100 mm horizontal line with two anchors, where zero represents ‘no pain at all’, and 100 represents ‘the most severe pain’. It has been shown to be a valid and reliable method for evaluating pain levels. [37].

To determine whether participants feel that they have benefited from the intervention, global perceived effect (GPE) will be measured with the GPE scale. [38] This is a 7-point ordinal scale, ranging from “completely recovered” to “worse than ever”.

The effect of the exercise programs on QOL will be assessed with the 12-item Short Form Health Survey (SF-12) [39] and the Euroqol instrument (EQ-5D) [40] during baseline and follow-up measurements.

To assess functional performance of the muscle-tendon unit, two different tests with acceptable reliability (intraclass correlation coefficients 0.78–0.91) will be used. Firstly, participants will perform the heel-raise test, [41] which is recommended for the evaluation of calf muscle endurance in patients with AT. [42] Participants are asked to stand on one leg with a straight knee, supporting with their fingertips to the wall for balance. They are asked to perform as many heel raises as possible, with a straight knee and a frequency of one heel raise every 2 s, avoiding forward body sway. The test is terminated when the participant stops, cannot keep the frequency, or when the technique is incorrect for two consecutive repetitions. The total number of heel raises will be used for data analysis. Secondly, the one-legged countermovement jump (CMJ) will be used to evaluate jump height. [42] This test has previously been used as a functional outcome measure in patients with midportion AT. [25] Although jump height is determined by many other muscle groups, research has shown that the calf muscle complex accounts for an important part of the CMJ movement. [43].

The CMJ is performed with the participant in an upright position on a jumping platform (Projump, Biometrics, The Netherlands), with the hands placed behind the back. Participants are asked to quickly bend their knee as much as they want and then immediately jump upwards to their maximum height. They are allowed three maximal trials, and the best jump height (in cm) is used for data analysis. Pain during the heel raise tests and CMJ is recorded on a 0–10 numerical pain rating scale.

Differences in cost-effectiveness between both programs will be investigated by collecting several variables that are related to direct and indirect (medical) costs during follow-up assessments. These costs include medical consumption (visits to healthcare providers, supplementary diagnostics such as imaging, additional therapies such as insoles, braces, and medication use), and injury related absenteeism from (un)paid work, school, and sport.

#### Other outcome measures

Morning stiffness is common in patients with AT, and is considered a good indicator of tendon recovery. [3] Participants will rate their morning stiffness (in minutes) in a logbook. This logbook is also used to record compliance to the exercise program. Compliance will be calculated by dividing the amount of exercises actually performed by the prescribed amount of exercises (i.e., 2× per day for the Alfredson group and 1×/day for the Silbernagel group). Subsequently, compliance will be categorized into four categories: poor (< 25%), moderate (between 25 and 50%), good (between 50 and 75%) and excellent (> 75%). [28].

Furthermore, participants will record other (medical) treatments and medication use in the logbook.

At baseline and follow-up measurements, the isometric strength of the hip extensors, abductors, and external rotators will be measured using a handheld dynamometer, according to previously reported methods. [44] Male patients with AT demonstrate diminished strength of their hip musculature compared to asymptomatic controls, [44] and by evaluating hip muscle strength over the course of this study, we try to investigate whether this is a prognostic factor in patients with AT.

### Measurements

All measurements will be conducted at baseline (T0), at 12 weeks (i.e. termination of intervention, T1), 26 weeks (T2), and at 52 weeks (T3) follow up, and include both the afore mentioned questionnaires and physical examination. Participants can complete the questionnaires online (secured environment), by using a hyperlink that will be sent to them by e-mail. All physical assessments are conducted by the same investigator (BH), who is blinded to group allocation. For a detailed overview of all outcome measures collected in the course of the study and the respective follow-up times, see Table 2.

**Table 2:** Overview of outcome measures collected in the course of the study

	*Baseline*	*12 weeks*	*26 weeks*	*52 weeks*
Eligibility criteria check	*X*			
Body height	*X*			
Body weight	*X*	*X*	*X*	*X*
BMI	*X*			
Job type & activity level	*X*			
Sport type & activity level	*X*	*X*	*X*	*X*
Referral type	*X*			
Waist circumference	*X*			
Dorsiflexion ROM ankle	*X*			
Dorsiflexion ROM first MTPJ	*X*			
VISA-A score	*X*	*X*	*X*	*X*
VAS for pain during sport and daily activities	*X*	*X*	*X*	*X*
Morning stiffness	*X*	*X*	*X*	*X*
Global perceived effect (7-point scale)	*X*	*X*	*X*	*X*
Quality of life (SF-12 and EQ-5D)	*X*	*X*	*X*	*X*
Functional performance (CMJ and heel raise test)	*X*	*X*		*X*
Variables related to cost-effectiveness: - Medical consumption - Absenteeism from (un)paid work - Absenteeism from school - Absenteeism from sports activities	*X*	*X*	*X*	*X*
Compliance to exercise program		*X*		
Isometric strength of hip musculature	*X*	*X*		*X*

*BMI* body mass index, *ROM* range of motion, *MTPJ* metatarsophalangeal joint, *VISA-A* Victorian Institute of Sports Assessment – Achilles, *VAS* visual analog scale, *SF 12* 12-item short from health survey, *EQ-5D* Euroqol instrument, *CMJ* countermovement jump

### Statistical analyses

Differences between the Alfredson and Silbernagel group will be analyzed according to intention-to-treat (ITT) principle. If necessary, missing data will be imputed using multiple imputation. Descriptive statistics will be calculated for all continuous variables, and means and SDs will be reported (or median and interquartile range for non-parametric data). For nominal and categorical data, proportions will be calculated and reported.

Baseline comparability of the two groups will be assessed by the Student t-test (parametric data), and non-parametric tests where appropriate. To assess differences in (pseudo)metric data within and between the groups over time, an analysis of variance (ANOVA) will be performed, with post-hoc tests to correct for multiple testing. Multivariate regression techniques will be conducted to model the prognostic value of baseline variables on outcome. The cost-effectiveness will be estimated by calculating the incremental cost-effectiveness ratio: (costs of Silbernagel program – costs of Alfredson program) / (health benefit of Silbernagel program – health benefit of Alfredson program), and will be expressed as costs per quality adjusted life year (QALY).

All analyses will be performed with statistical significance level set at α = .05 (two-sided).

## Discussion

Several studies showed that both Alfredson isolated eccentric and Silbernagel combined concentric-eccentric training are beneficial in terms of symptom reduction in midportion AT. [14, 16, 17, 25, 26] However, whether any of these programs is more effective has yet to be determined. This study protocol describes the first RCT directly comparing the effectiveness of both programs. We designed a pragmatic study, in which we try to replicate how both programs are described in the clinical setting. By using the VISA-A – a condition-specific validated questionnaire that is widely recommended for use in research and clinical practice – as the primary outcome measure, we hope that comparison of our results to other studies and clinical practice will be enabled.

Besides the effectiveness on symptom reduction and QOL, our study also compares the effectiveness of both programs on functional performance of the muscle-tendon unit. This comparison has not previously been performed, whilst research has shown that functional deficits of the muscle-tendon unit may still persist after 1 year in patients with AT, even though symptoms have fully recovered. [45].

We will also assess differences in cost-effectiveness in the mid-term and long term. Cost-effectiveness may be an important parameter for clinical decision making, but to date, research investigating cost-effectiveness in AT treatment is scarce. [17] We expect no difference in direct intervention-related costs, since both programs are performed at home and the amount of supervising physiotherapy sessions is similar, but we are predominantly interested in potential differences in indirect costs (e.g. absenteeism of work, school and sports) between both programs.

Recruitment for this trial will be performed in different institutions, i.e. sports medicine clinics, hospitals, and general practices. Therefore, participant characteristics may differ, and this potentially could lead to different subgroups of participants. No stratified randomization for referral type is performed, but by including recreational athletes, it is expected that both study arms will consist of relatively homogeneous groups. Furthermore, we try to collect important participant characteristics that may cause potential bias to the results of this study. Nonetheless, it should be acknowledged that the participants’ metabolic health is not fully covered, whilst research showed that this may be a confounding factor. [46].

We feel that the pragmatic nature of our study is a strength, as it mimics the clinical setting. Nevertheless, a potential limitation of this pragmatic design that we cannot draw any conclusions on the underlying mechanism of possible differences in effectiveness, since we have not controlled for many of the potential contributing factors.

Additionally, our study does not include a study arm performing no exercise intervention. Therefore, it remains unknown whether potential improvements are caused by the exercise programs or by the natural course of the condition. Although it is generally agreed that exercise therapy is crucial in the treatment of AT, [3, 9, 12] we acknowledge that the lack of a non-exercise (wait-and-see) group is a potential limitation of the study design.

In summary, this multicenter two-arm RCT will compare the effectiveness of the Alfredson isolated eccentric to the Silbernagel combined concentric-eccentric program for treatment of chronic midportion AT. The results of this study will enlarge the evidence base on different exercise programs for AT, and may aid the clinician in choosing the most appropriate program for their patients.

### Trial status

Enrollment of participants has started since November 2016. On July 3 2017, 14 participants have been included.

## Abbreviations

ANOVA: analysis of variance
AT: Achilles tendinopathy
CMJ: countermovement jump
EQ-5D: Euroqol instrument
GPE: global perceived effect
HSRT: heavy slow resistance training
ITT: intention-to-treat
QOL: quality of life
RCT: randomized controlled trial
SF-12: 12-item short form health survey
UMCU: University Medical Center Utrecht
VAS: visual analog scale
VISA-A: Victorian Institute of Sports Assessment – Achilles
